# The INDDEP study: inpatient and day hospital treatment for depression – symptom course and predictors of change

**DOI:** 10.1186/1471-244X-13-100

**Published:** 2013-03-26

**Authors:** Almut Zeeck, Joern von Wietersheim, Heinz Weiss, Manfred Beutel, Armin Hartmann

**Affiliations:** 1 Department of Psychosomatic Medicine and Psychotherapy, Medical University Hospital, Freiburg, Germany; 2 Clinic for Psychosomatic Medicine and Psychotherapy, Medical University Clinic, Ulm, Germany; 3 Department for Psychosomatic Medicine and Psychotherapy, Robert-Bosch-Krankenhaus, Stuttgart, Germany; 4 Department of Psychosomatic Medicine and Psychotherapy, Medical University Hospital, Mainz, Germany

**Keywords:** Depression, Inpatient, Day hospital, Predictors, Naturalistic study, Outcome, Subgroups

## Abstract

**Background:**

Depression can be treated in an outpatient, inpatient or day hospital setting. In the German health care system, episodes of inpatient or day hospital treatment are common, but there is a lack of studies evaluating effectiveness in routine care and subgroups of patients with a good or insufficient treatment response. Our study aims at identifying prognostic and prescriptive outcome predictors as well as comparative effectiveness in psychosomatic inpatient and day hospital treatment in depression.

**Methods/Design:**

In a naturalistic study, 300 consecutive inpatient and 300 day hospital treatment episodes in seven psychosomatic hospitals in Germany will be included. Patients are assessed at four time points of measurement (admission, discharge, 3-months follow-up, 12-months follow-up) including a broad range of variables (self-report and expert ratings). First, the whole sample will be analysed to identify prognostic and prescriptive predictors of outcome (primary outcome criterion: Quick Inventory of Depressive Symptoms QIDS-total score, expert rating). Secondly, for a comparison of inpatient and day hospital treatment, samples will be matched according to known predictors of outcome.

**Discussion:**

Naturalistic studies with good external validity are needed to assess treatment outcome in depression in routine care and to identify subgroups of patients with different therapeutic needs.

**Trial registration:**

Current Controlled Trials ISRCTN20317064

## Background

Depression is one of the most common diseases with a life time prevalence rate of 17.1% and a 12-months prevalence of 10.7% [1]. It is a leading cause of disability worldwide [2]. The German health care system provides outpatient, inpatient and day hospital care for depression [3]. Two medical specialties are involved in the treatment of mental disorders: psychiatry and psychosomatic medicine. Psychosomatic inpatient and day hospital treatment programs are characterized by a stronger focus on psychotherapy as the main treatment modality, integrating psychopharmacological and somatic treatment elements. About 40-50% of the patients treated in psychosomatic hospitals have a main diagnosis of major depression [4-6]. In contrast to many other countries, inpatient treatment plays an important role: ~64.500 beds are provided in psychiatric and ~6400 in psychosomatic hospitals for acute care, with an additional 15.400 beds in psychosomatic rehabilitation hospitals, covering a considerable part of health care for people with mental diseases.

In comparison to outpatient treatment, hospital programs have the advantage of using a multimodal approach, combining daily structure with individual, group and additional treatment components [5]. Day hospital programs for acute psychosomatic care are comparable to inpatient programs with the difference that patients return home at evenings and weekends. There is an increasing interest in day hospital programs, because of the lower costs of this treatment modality [7,8].

Although treatment of depression is a high priority task in psychosomatic medicine as well as in psychiatry, there is a lack of studies on inpatient or day hospital treatment. Additionally, previous studies do not take into account the marked heterogeneity of patients with this diagnosis: Depression in one subject is not like depression in another (“uniformity myth”; [9]). This will be especially true for the patient group treated in inpatient and day clinic units, who show high rates of co-morbidity with mental or somatic disorders, a severe course of the illness or complex problems including the social and family situation. It can be postulated that tailoring treatments to the needs of subgroups of patients with special characteristics will improve overall outcome, which is reported to lie around a mean value of 30-40% remission rates after “standard treatment” for depression [3].

There are few naturalistic studies describing effects of psychosomatic inpatient and day hospital treatment, mostly lacking follow-up assessments and a detailed description of patients characteristics (personality, co-morbidity) [4-6,10-12]. The majority of studies report on heterogeneous samples including depressed patients besides other diagnoses. There is no study trying to identify subgroups of patients with a main diagnosis of major depression with different symptom courses and no study comparing inpatient and day hospital treatment. The same is true for psychiatry, with a limited number of studies on this topic conducted in the German health care system (see for example: [13-15]).

The following predictors were repeatedly found to be associated with the course and overall outcome in depression: (1) number of episodes of major depression (MDE), (2) duration of the current MDE, (3) co-morbidity (mental, somatic) and (4) incomplete remission of the last episode of MDE or “double depression” [3]. A higher probability for a relapse is associated with younger age, female gender, a lack of social support and being single. An additional somatic illness and a late onset of treatment are able to nearly completely explain prognosis in a higher age group [16]. Traumatic childhood experiences were also identified as a risk factor for depression and important to consider in terms of the long term course and risk for relapse [17]. Most of these factors have been found in studies on outpatient treatment for depression. It remains unclear, if the same predictors will be identified analysing samples of patients treated within an inpatient or day hospital setting.

Another group of studies focusses on the influence of personality characteristics on the course and prognosis of depression. One of the most relevant concepts in this field is the one of Sydney Blatt, who postulated a two-dimensional model, which differentiates between depressed patients who primarily deal with problems in interpersonal relatedness (“anaclitic”: fears of abandonment; dependency) and those preoccupied with self-definitional issues (“introjective”: hash self-criticism; perfectionism) [18]. There is an ongoing debate on the relevance of this theoretic model^a^. In support of it, perfectionism as a trait was repeatedly found to be associated with a more problematic course in depression. Overall, it can be assumed that personality traits and other patient variables (social support, interpersonal problems, etc.) are not only overall predictors of outcome, but also interact with the form of treatment or setting provided.

Studies comparing inpatient and day hospital treatment found comparable outcomes for patients with mental disorders, for whom both settings are suitable [8]. We could replicate this finding for a psychosomatic clinic in Germany [11], but found differences in effects when analysing outcome for single diagnostic categories like eating disorders [19,20]. Comparing means of large samples might cover differences between subgroups, which can be better treated in one or the other setting. In a pilot study, we tried to identify predictors of differential outcome of inpatient and day hospital treatment and found a higher motivation for psychotherapy and higher burdens at home to be associated with a good outcome after day hospital treatment, whereas strong wishes to give up responsibility for oneself were associated with an unfavourable course of inpatient treatment [5,21]. Strong wishes to give up responsibility might be associated with “anaclitic” features according to S. Blatt et al. [18], but this has to be shown empirically.

The INDDEP (**In**patient and **D**ay Hospital Treatment for **Dep**ression) - study is a naturalistic study which aims at the description and comparison of symptom courses after psychosomatic inpatient and day hospital treatment for major depression. It further aims at identifying “prognostic” (associated with general outcome) and “prescriptive” (associated with the differential outcome in both settings) variables, which can help to discriminate subgroups of patients with differences in course and treatment needs [22]. Finally, we aim to go beyond previous studies in assessing not only socio-demographic variables and aspects of depressive symptomatology, but also personality traits, coping style and interpersonal problems as possibly relevant predictors.

## Methods

For to analyse a sample which represents the clinical reality of psychosomatic inpatient and day hospital treatment for depression in Germany (external validity), we did not choose a randomized design as we expected only a minority of patients to agree to a randomization procedure and expected considerable resistance in non-university centres. Therefore, the study is naturalistic in nature and composes two steps: I) an analysis of predictors in the whole sample and II) a comparison of inpatient and day clinic treatment in a quasi-experimental design. For a comparison of inpatient and day clinic treatment, samples will be parallelized according to known predictors of outcome (see: methods against bias).

### Study centers

The study is carried out at 7 German study sites. These comprise three psychosomatic departments at university hospitals and four clinics outside the university context. All departments or clinics provide a treatment program that is typical for clinics of psychosomatic medicine in Germany (see [5,21]). Participating centers are the Department of Psychosomatic Medicine and Psychotherapy at the University Clinic of Freiburg, the Clinic of Psychosomatic Medicine and Psychotherapy at the University of Ulm, the Department of Psychosomatic Medicine and Psychotherapy at the University Clinic of Mainz, the Clinic for Psychosomatic Medicine and Psychotherapy at the Robert-Bosch-Krankenhaus Stuttgart, the Thure-von Uexküll-Klinik Freiburg, the Clinic for Psychosomatic Medicine and Psychotherapy at the Bürgerhospital in Stuttgart and the Rhein-Klinik, Bad Honnef.

### Participants

During a 2.5-year recruitment period, all patients admitted to the participating clinics will be screened for depressive symptoms and inclusion and exclusion criteria checked. Recruitment will be continued until a sample of 300 patients is reached in each setting (inpatient, day hospital). There has to be a minimum of 35 patients per setting in each centre to control for centre effects. Research assistants will be involved, if a depression can be identified as the main reason for admission or the most dominant problem (main diagnosis). The research assistant will inform the patient about the study and check inclusion and exclusion criteria. Inclusion and exclusion criteria are listed in Table 1. Patients will get comprehensive information about the study and are included after written consent. There is permission from the local ethics committees.

**Table 1:** Inclusion and exclusion criteria of the INDDEP-study


Inclusion criteria	• MDE (major depressive episode, unipolar, according to DSM IV) as main diagnosis
	• age 18-65
	• QIDS-C score > 10
	• informed consent
	• sufficient knowledge of German language
Exclusion criteria	• psychotic disorder (current or life time)
	• bipolar disorder
	• substance dependency (current or last three years)
	• current suicidal ideation
	• antisocial personality disorder
	• cognitive impairment, dementia
	• admission for diagnostic reasons (not for treatment)
	• second admission during recruitment period

### Interventions

Interventions comprise the routinely provided inpatient and day hospital treatment programs of psychosomatic clinics in Germany. These include individual and group psychotherapy, art and body therapy, relaxation therapy, family sessions and support of a social worker if needed, psychopharmacology, educational elements, physician rounds and medical care (see [5]). Treatment duration usually comprises between 4 and 12 weeks [5].

### Measurement time points

Time points of measurement will be the time point at admission (T0), at discharge (T1), a three months follow-up (T2) and a 12 months follow-up (T3). Data will include a detailed assessment on different levels: symptomatology (characteristics of depression and co-morbid conditions), general disturbance and quality of life, aspects of personality, interpersonal problems and a further aspects that has been identified to influence the course of depression: traumatic childhood experiences. Variables will be grouped into classes of possible predictors. A combination of self-report and assessments by independent, trained research assistants will be used (see Table 2).

**Table 2:** Instruments used the INDDEP study

**Time point of measurement**	**Outcome/Variables**	**Patient**	**Research assistant**
		**(self-report)**	**(expert rating)**
**T0 **(admission)	Depressive symptomatology:	QIDS-SR	QIDS-C, SKID-I
	Co-morbidity		SCID I + II
	Personality, interpersonal problems, mode of coping:	IIP-32, DEQ, DAS	
	Overall disturbance and functioning:	SCL-90-R	SOFAS
	Social support:	F-SozU K-14	
	Quality of Life:	SF-12	
	Traumatisation:	CTQ	
**T1 **(discharge)		QIDS-SR	QIDS-C
		SCL-90-R	SOFAS
		IIP-32	
**T2 **(3-months Follow-up) and **T3 **(12-months Follow-up)		QIDS-SR	QIDS-C, LIFE-Interview
		SCL-90-R	SOFAS
		SF-12	
		IIP-32	

QIDS-SR = Quick Inventory of Depressive Symptoms, self-report form [23], QIDS-C = expert rating; SCID-I and SCID-II=Structured Clinical Interview for DSM IV axis I and II [24,25]; SCL-90-R = Symptom-Check-List 90-R [26]; DAS = Dysfunctional Attitudes Scale [27,28]; DEQ = Depressive Experiences Questionnaire [29,30]; SOFAS = Social and Occupational Functioning Assessment Scale [31]; IIP-32 = Inventory of Interpersonal Problems, 32-item version [32,33]; F-SozU K-14 = Fragebogen zur sozialen Unterstützung, 14-item version [34]; SF-12 = Fragebogen zum Gesundheitszustand (quality of life) [35]; CTQ = Childhood Trauma Questionnaire [36,37]; LIFE-Interview = Longitudinal Interval Follow-up Evaluation [38].

Note: Additionally assessed at all time points of measurement: pharmacological treatment, somatic diagnoses; at T0: previous treatment for depression; at T1: documentation of treatment received (dose, components); T2 and T3: further outpatient treatment, days of sick leave, number of contacts to health care system.

### Objectives and hypotheses

First, the study aims to describe changes in depressive symptomatology during and after psychosomatic inpatient and day hospital treatment for depression and to identify subgroups with a good or less favourable symptom course (observational) including follow-up assessments.

Secondly, inpatient and day clinic treatment will be compared (matched samples). It is assumed that type of depression according to S. Blatt (introjective/high level of perfectionism vs. anaclitic/high level for dependency) is associated with differential outcome in each setting.

### Outcomes

The Quick Inventory of Depressive Symptomatology QIDS-C (expert rating) is chosen as the main outcome criterion. The QIDS is a 16-item version of the Inventory of Depressive Symptomatology (IDS), which shows high correlations with other instruments for the measurement of depressive symptomatology [23]. It´s a common, internationally used, licence-free instrument which has a self-report and an expert rating form [23]. The QIDS was chosen as its total score is also used as the main outcome criterion in a large trial on outpatient psychotherapy for chronic depression that is currently conducted in Germany (“LAC-study”, [39]).

We aim to assess outcome dimensionally (change on the QIDS-C score from admission to the three-months follow-up) as well as categorically: a symptom reduction < 20% is defined as “no effect”, a symptom reduction between 20% and 50% as modest change, a symptom reduction of > 50% as partial remission and a symptom reduction of 100% as complete remission (falling below the cut-off value of depression of the QIDS-C) [3,40].

### Sample size calculation

The power calculation is based on assumptions about differences in the frequencies of remissions by treatment setting, the difference of means on the outcome measure, attrition and loss of cases due to mismatches. Given the probability of remission was p=0.30 for treatment A and p=0.45 for treatment B, then for equal *N* in each group, α = .05 and (1-β) = 0.80, the required sample size is *N* =180 (each group; calculated with PASS2008). For a standardized difference of *d* = 0.2 (small effect size) and α = .05 and (1-β) = 0.80, the required sample size is *N* = 200. With an attrition rate of 10% and loss due to mismatches of 20% (matching samples for research question II), the total sample size should be increased to *N* = 300 per group. Including covariates, subgroups and nesting (HLM) will help to increase the precision of models.

### Methods against bias

To compare inpatient and day clinic samples, a parallelization according to known predictors of outcome [14] will be conducted: (1) Gender [m;f], (2) age [<40; >=40: mean age in previous studies], (3) number of additional axis-I diagnoses [<2; >=2], (4) number of previous episodes of major depression [0; <=2; >2] and (5) duration of the recent episode of MDE [<6 months; 6–24 mo >24 mo]. Criteria (1) to (3) will be matched 1:1; criteria (4) & (5) are used as lenient criteria, to be matched 1:1 if possible, otherwise the extremes may not be matched together. We expect a loss of about 20% due to “mismatches”, missing data and patients dropping out of the study.

Antidepressant medication, the initial score of the QIDS-C and the number of psychotherapy sessions following discharge will be controlled for in the final analysis. At the end of the study, patients screened and not taking part and patients taking part in the study will be compared, as well as patients dropping out of the study with those completing the study.

### Statistical analysis

The statistical analysis will follow the example of Fournier et al. [22]. The continuous outcome (QIDS-C) will be analyzed with hierarchical linear models (HLM; nested random effects). The difference (T0-T2) will be controlled for baseline (T0) severity. Moderator and mediator analyses will be conducted according to Kraemer et al. [41]. The analysis of categorical outcome (remission classes) will be conducted with categorical regression with methods of optimal scaling (SPSS-CatReg) [42]. The identification of predictors with SPSS-CatReg is based on boot-strapping procedures or on the “Least Absolute Shrinkage and Selection Operators” [42]. For the HLM we will adapt the example of Fournier et al. [22] restricting the degrees of freedom of the selection of predictors in a hierarchical process.

### Safety aspects and medical complications

There is a pseudonymisation of the data. At the end of the study, the data per centre will be made available to the participating centres who will remain owners of their own data sets. The coordinating centre (Freiburg) is responsible for data integrity and monitoring. Selected adverse events (like suicide attempts, transfer to other departments because of somatic problems or suicidal ideation) are defined in the study protocol, monitored and followed up constantly throughout the study and documented accordingly by the investigator including evaluation. Serious adverse events and other safety issues that might materially alter the current risk-benefit assessment should be reported by the coordinating investigator (A.Z., Freiburg) and to the responsible ethics committee according to the study protocol. As all patients get the usual standard treatment of routine care (observational study), criteria for ending the study because of adverse events are not applicable.

### Ethical issues

The final study protocol and the final version of the written informed consent form were approved by the ethics committee of the University of Freiburg as well as the local ethics committees. The trial will be carried out according to legal and regulatory requirements, the principles of good clinical practice and according to the Declaration of Helsinki.

## Discussion

The INDDEP study is a naturalistic study, which aims at the description of symptom courses in patients with depression receiving inpatient or day hospital treatment in psychosomatic clinics in Germany and the identification of subgroups by analyzing prognostic and prescriptive variables. Secondly, it aims at comparing effects of inpatient and day clinic treatment using a quasi-experimental design (matched samples).

A naturalistic design and broad inclusion criteria were chosen to achieve high external validity of data and to describe treatment and symptom courses in routine care. A randomized trial comparing inpatient treatment with outpatient or day clinic treatment would have led to a highly selective sample of patients having no treatment preference. It would have resulted in data on efficacy of treatments, but only in a small subsample. For a comparison of settings we choose to use the next best design: Inpatient and day hospital samples will be parallelized according to known predictors of outcome [14]. In the power calculation we took into consideration that sample size will be reduced by this procedure.

The INDDEP study is the largest study on inpatient and day clinic treatment in depression in the German health care system. In comparison to previous studies, it has the advantage that patients are diagnosed by structured interviews and followed up 3 and twelve months after discharge by self and expert assessments. Measurement is not limited to symptom change only, but comprises the evaluation on different levels relevant for overall functioning and quality of life: socio-demographic data, co-morbidity (mental, somatic), personality, coping strategies, interpersonal problems, traumatization and social support. Additionally, there will be a comprehensive documentation of the utilization of health care services after discharge, as well as a documentation of days of work absenteeism allowing an economic analysis.

To study inpatient and day hospital treatments can be seen as a problematic task, as treatments consist of multiple therapeutic elements, are provided by different treatment teams and hospitals are embedded in different environments. We can only try to reduce bias as good as possible. To control for centre effects, each hospital has to recruit a minimum of 35 patients in each setting. Treatment components will be documented in detail (sort, duration, frequency), although in a pilot study we found treatment programs to be quite comparable in dose and composition of elements, due to German standards for psychosomatic clinic programs. We will take the nesting of data (settings, clinics) into consideration by using complex statistic methodology. But we cannot rule out that the fact of taking part in a study influences treatment course and has some sort of impact.

In terms of dropping out of the study, we expect rates to be low, as there are much more advantages for agreement to participate compared to a refusal: Patients will get in-depth diagnostic assessments and allowances for filling in the questionnaires or taking part in follow-up assessments (20€ for each time point of measurement). Furthermore, depressed patients usually are a group of patients with a comparably high compliance rate.

The principal investigators and participating centers are experienced in the conduction of studies and all hospitals provide the usual standard care of psychosomatic centres. Most of the hospitals have been involved in the preceding pilot study [5,21].

Overall, we think this study will provide important data on the treatment of depression in the German health care system and help to tailor treatment programs to the needs of different subgroups of patients.

## Endnotes

^a^See for example: Psychotherapy Research, volume 20, number 1^st^ of January 2010.

## Abbreviations

HLM: Hierarchical linear models; MDE: Major depressive episode; QIDS: Quick Inventory of Depressive Symptoms.

## Competing interests

The authors declare that they have no competing interests.

## Authors’ contributions

AZ is the principal investigator of the study, JvW is the co-investigator. AH is the leading biostatistician. HW made essential contributions to development and design of the study. MB made substantial contributions to methodology and drafted sections of the manuscript. All authors read and approved the manuscript.

## Pre-publication history

The pre-publication history for this paper can be accessed here:

http://www.biomedcentral.com/1471-244X/13/100/prepub
